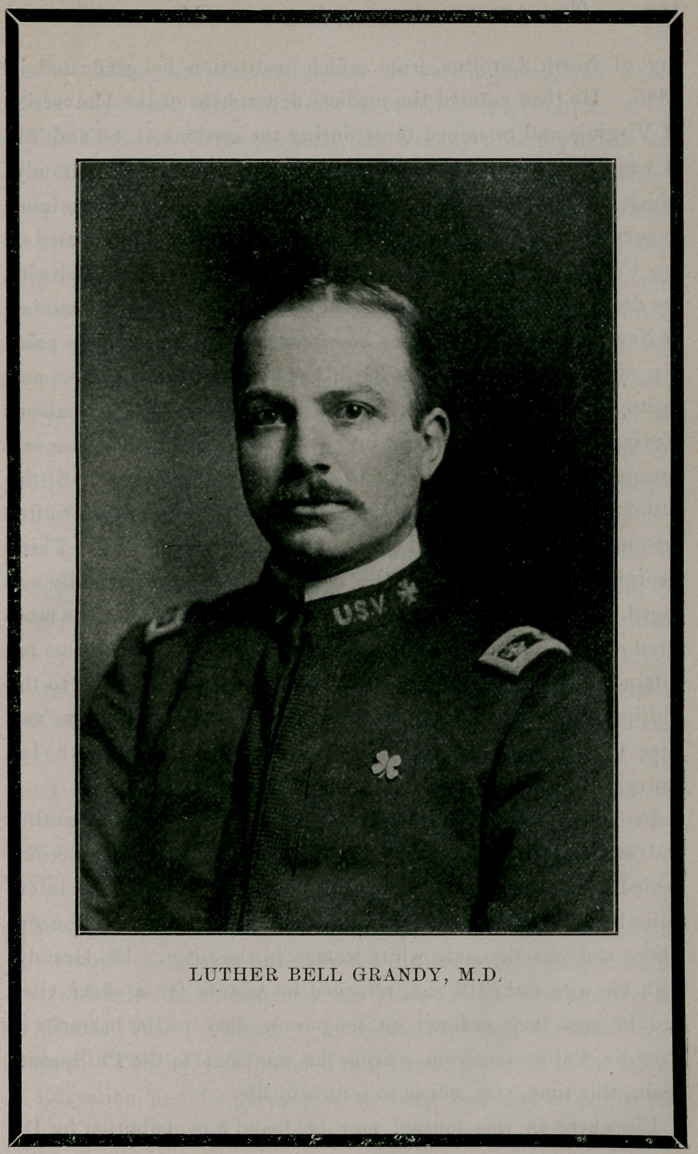# Luther Bell Grandy

**Published:** 1902-06

**Authors:** 


### EDITORIAL NOTES AND COMMENTS.

The Business office of THE JOURNAL-RECORD is No. 64 Marietta Street.

The Editorial office is 318-319-320 The Prudential.

Address all Business communications to Dr. M. B. Hutchins, Mgr.

Make remittances payable to THE ATLANTA JOURNAL-RECORD OF MEDICINE.

On matters pertaining to the Editorial and Original Communications address Dr. Bernard Wolff, Atlanta.

Reprints of original articles will be furnished at cost price. Requests for the same should always be made *on the manuscript*.

We will present, post-paid, on request, to each contributor of an original article, twenty (20) marked copies of THE JOURNAL-RECORD containing such article.

### LUTHER BELL GRANDY.

IN the last issue of this journal it was our painful task to announce the sudden death from apoplexy of our colleague, Dr. L. B. Grandy. Dr. Grandy at the time of his death was serving in the capacity of major and surgeon of the 35th Regiment of Infantry, and was stationed at Lipa, in the Philippine Islands. His death was totally unexpected, for when he was last in Atlanta the improvement in his general health during his residence in the Philippines was very marked. It is sad that so much good blood and useful lives should be sacrificed to retain possession of a remote and doubtfully valuable territorial acquisition. The details of Dr. Grandy's death are exceedingly meagre, merely a brief cablegram from his wife announcing the shocking intelligence and confirmation of it from the Surgeon-General's office.

Luther Bell Grandy was the son of Titus T. and Elizabeth Bell Grandy. He was born at Oxford, N. C., April 3, 1865, and was therefore just 37 at the time of his death. He attended the Horner School at Oxford, N. C., where he was prepared for the Univer-

LUTHER BELL GRANDY, M.D.

sity of North Carolina, from which institution he graduated in 1885. He then entered the medical department of the University of Virginia and remained there during the sessions of '86 and '87. It was there that the writer made the acquaintance of Dr. Grandy, which ripened into close friendship in after years. Determining to complete his medical course in New York, he matriculated in the College of Physicians and Surgeons and was graduated with the degree of doctor of medicine in June, 1890. After remaining in New York for a short time he removed to Atlanta for the practice of his profession. Having always displayed a fondness and aptitude for literary work he soon drifted into medical journalism. Having purchased an interest in *The Atlanta Medical and Surgical Journal* he became its editor, and continued in that office until the outbreak of the Spanish-American war. Within a short time after the outbreak of hostilities he was appointed surgeon of the Third Georgia Infantry. Though this regiment was never actually engaged, it saw some service in Cuba. When the regiment was mustered out Dr. Grandy returned to Atlanta, but in a short time re-entered the army as contract surgeon and was sent on duty to the Philippines. He was afterwards promoted to a captaincy, and later to the rank of major and surgeon of the Thirty-fifth Infantry, U. S. V.

In 1894 Dr. Grandy married Miss Hattie Smart, a beautiful and accomplished young lady of Atlanta. Mrs. Grandy accompanied her husband to the Philippines, and has written very interestingly of her life in the remote portion of the island of Luzon, where she was the only white woman in the town. Dr. Grandy, with his wife and little son, returned to Atlanta for a short visit, and he was then ordered on temporary duty to the barracks at Presidio, California, from whence he was sent to the Philippines again, this time, alas, never to return in life.

Elsewhere in this journal may be found a contribution by Dr.

Grandy which was destined never to be completed, and which would have been without doubt a valuable contribution to the slender literature of the subject. Dr. Grandy's literary work was of a high character, and was marked by a strong and thorough grasp and familiarity with every subject upon which he expressed his opinion. He had the mental bent of the historian, the ability to grasp and set forth the strong points of a subject in simple and convincing terms. As editor of this journal, his editorial utterances were frequently copied by reason of their timeliness and force. His paper on the history of anesthesia has become classic, and probably no man up to his time so patiently searched out the data and so truthfully and dispassionately presented them to the public. The result of his research was an overwhelming confirmation of the priority of Crawford W. Long in his contention. Dr. Grandy also prepared a brief medical history of the State of Georgia which will be referred to doubtless as authoritative for all time. Dr. Grandy's personality was extremely attractive. Mild and gracious in manner, there was an undertone of force and strength. As a physician he was accomplished, cool in judgment and accurate in therapeutics; as a friend he was steadfast and loyal. Impressed forever upon the hearts of a multitude of earnestly devoted friends are the generous, noble and manly qualities of their departed companion, and their sympathies go out in the hour of heavy affliction to the wife and little son who are left to mourn his loss.

---

THE physicians appointed as medical visitors of the public schools of Atlanta have effected a permanent organization, of which Dr. L. P. Stephens was elected President and Dr. A. G. Hobbs Vice-President. A constitution and by-laws were adopted. This body is intended to act as an adjuvant to the Board of Education, and its purpose is to keep under observation the general hygiene and sanitation of the school buildings, and the public school pupils, and to offer at times such recommendations as are deemed necessary with regard to these conditions.

In many instances the buildings are in an unsanitary state and it seems likely that the association will be of material service in securing from the City Council the funds necessary to carry into effect the desired improvements during the coming vacation.

---

THE Fourteenth Annual Meeting of the Tri-State Medical Society of Alabama, Georgia and Tennessee, will be held at Birmingham, Ala., October 8, 9 and 10, 1902. This meeting promises to be of unusual interest from present indications. One of the prominent features of the last meeting, and which attracted considerable attention, was the discussion of sociological questions. The papers in this section excited considerable comment locally in many of the journals throughout the country.

---

DR. GEORGE BROWN, of Atlanta, proposes another world's congress on tuberculosis, to be held at St. Louis during the Louisiana Purchase Exposition in 1904. Such gatherings are productive of good, and certainly nothing is more needed than organized efforts at the repression of one of the prime menaces of human existence. The profession generally should take this matter in hand and cooperate to the materialization of the proposed conference.

---

DR. GILMAN P. ROBINSON died at his residence in Atlanta on May 27. He was 46 years of age. Dr. Robinson was a native of Rhode Island and a graduate of Harvard Medical College. He removed to Atlanta in 1899 and confined his practice to diseases of children, and was a lecturer on that subject in the College of Physicians and Surgeons. He was held in much esteem and regard and his death is greatly to be deplored.

---

ATTENTION is called to the forthcoming meeting of the Georgia Sociological Society. The objects of the society are eminently praiseworthy and much public interest is being aroused in its work. Membership is open to all who are interested in its purposes.

## Figures and Tables

**Figure f1:**